# A new species of *Princaxelia* from Shinkai Seep Field, Mariana Trench (Crustacea, Amphipoda, Pardaliscidae)

**DOI:** 10.3897/zookeys.1015.59683

**Published:** 2021-02-04

**Authors:** Ko Tomikawa, Hiromi Kayama Watanabe, Katsuhiko Tanaka, Yasuhiko Ohara

**Affiliations:** 1 Graduate School of Humanities and Social Sciences, Hiroshima University, Higashi-Hiroshima 739-8524, Japan Hiroshima University Higashi-Hiroshima Japan; 2 X-STAR, Japan Agency for Marine-Earth Science and Technology (JAMSTEC), 2-15 Natsushima-cho, Yokosuka, Kanagawa 237-0061, Japan X-STAR, Japan Agency for Marine-Earth Science and Technology Yokosuka Japan; 3 Department of Marine Biology, School of Marine Science and Technology, Tokai University, 3-20-1, Orido, Shimizu, Shizuoka, Shizuoka 424-8610, Japan Tokai University Shimizu Japan; 4 Hydrographic and Oceanographic Department of Japan, 3-1-1 Kasumigaseki, Chiyoda-ku, Tokyo 100-8932, Japan Hydrographic and Oceanographic Department of Japan Tokyo Japan; 5 Research Institute for Marine Geodynamics (IMG), Japan Agency for Marine-Earth Science and Technology (JAMSTEC), 2-15 Natsushima-cho, Yokosuka, Kanagawa 237-0061, Japan Research Institute for Marine Geodynamics Yokosuka Japan; 6 Nagoya University, Furo-cho, Chikusa-ku, Nagoya 464-8602, Japan Nagoya University Nagoya Japan

**Keywords:** COI, deep sea, first record, hadal zone, *Princaxeliamarianaensis*, systematics

## Abstract

A new pardaliscid amphipod, *Princaxeliamarianaensis***sp. nov.**, is described from a single female captured at the Shinkai Seep Field, Mariana Trench, from a depth of 5,689–5,683 m. A key to species of *Princaxelia* is provided. This is the first species of *Princaxelia* to be described from the Mariana Trench, and the second report of this genus from this region.

## Introduction

The benthic amphipod genus *Princaxelia* Dahl, 1959 occurs in deep waters of the Pacific Ocean (Lörz 2010). To date, four species have been described: *P.abyssalis* Dahl, 1959 from 6,435–9,530 m in the Aleutian, Kurile-Kamchatka, Izu-Ogasawara, Yap, Japan, Philippine, Bougainville, and Kermadec Trenches (Kamenskaya 1981, 1997); *P.jamiesoni* Lörz, 2010 from 7,055–9,583 m in the Kurile-Kamchatka, Japan, and Izu-Ogasawara Trenches (Lörz 2010; Jażdżewska and Mamos 2019); *P.magna* Kamenskaya, 1977 from 7,190–7,250 m in the Yap Trench; and *P.stephenseni* Dahl, 1959, the type species of the genus, from 1,505 m off the coast of Iceland. *Princaxeliaabyssalis* and *P.jamiesoni* are reported to prey on other amphipods, suggesting that this genus is carnivorous (Jamieson et al. 2012).

The Shinkai Seep Field is a serpentinized, peridotite-hosted, cold-seep system which hosts an aggregation of chemosynthesis-based communities including *Abyssogena* clam, *Provanna* gastropod, and *Phyllochaetopterus* polychaete species. It is located northeast of the Challenger Deep, the deepest part of the Mariana Trench (Ohara et al. 2012; Okutani et al. 2013, 2016; Chen et al. 2018; Watanabe et al. in press). During one submersible dive on an expedition to this seep by R/V *Yokosuka*, a single specimen of a species referable to *Princaxelia* was collected. This is the first record of an identified *Princaxelia* species from the Mariana Trench. We here describe and illustrate this species as new.

## Material and methods

### Samples

**Figure 1.**
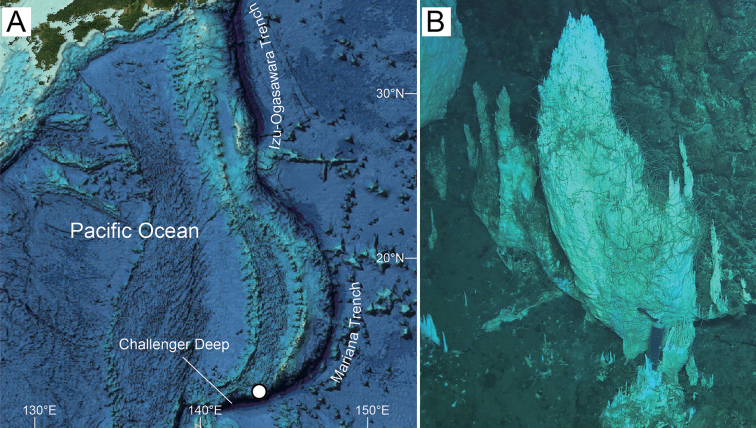
Sampling location and habitat of *Princaxeliamarianaensis* Tomikawa & Watanabe, sp. nov. **A** map indicating sampling location (circle) (map data from GEBCO Compilation Group [2020]) **B** sampling site at 5,686 m depth (Okumura et al. 2016).

The single *Princaxelia* specimen was collected from the Mariana Trench during dive 1402 of the deep-submergence vehicle (DSV) *Skinkai 6500* aboard R/V *Yokosuka* (cruise YK14-13, PI: Yasuhiko Ohara) by H. K. Watanabe (Fig. 1). Aboard the ship, the specimen was fixed and preserved in 99.5% ethanol. The specimen was sorted by K. Tanaka in the laboratory.

The holotype of *P.jamiesoni*, which was collected from the Japan Trench, was borrowed from the Tsukuba Collection Center of the National Museum of Nature and Science, Tokyo (NSMT-Cr 21250, female BL 56.2 mm), for comparison.

### Morphology

Appendages were dissected in 99% ethanol and mounted using gum chloral medium on glass slides with the aid of a stereomicroscope (Olympus SZX7). Appendages were examined by light microscopy (Nikon Eclipse Ni) and illustrated using a camera lucida. Body length (BL), from the tip of the rostrum to the base of the telson along the dorsal curvature, was measured to the nearest 0.1 mm. The only known specimen, the holotype, has been deposited in the collections of the American Museum of Natural History (**AMNH**).

### PCR and DNA sequencing

Genomic DNA was extracted from pereopod muscle of the holotype following procedures detailed in Tomikawa et al. (2014). The primer set for the cytochrome c oxidase subunit I (COI) gene (LCO1490 and HCO2198; Folmer et al. 1994) was used for the polymerase chain reaction (PCR) and cycle sequencing reactions. PCR and sequencing followed the methods detailed by Tomikawa et al. (2017). The DNA sequence has been deposited with the International Nucleotide Sequence Database Collaboration (INSDC) through the DNA Data Bank of Japan (DDBJ).

## Systematics

### Family Pardaliscidae Boeck, 1871

#### Genus *Princaxelia* Dahl, 1959

##### Princaxelia marianaensis

Taxon classificationAnimaliaAmphipodaPardaliscidae

Tomikawa & Watanabe sp. nov.

65A2D440-8966-5A55-9E0B-23CBBB3EB1E8

http://zoobank.org/B127A8B4-7BDA-4027-A7DA-8C04F61EA6BA

**Figure 2.**
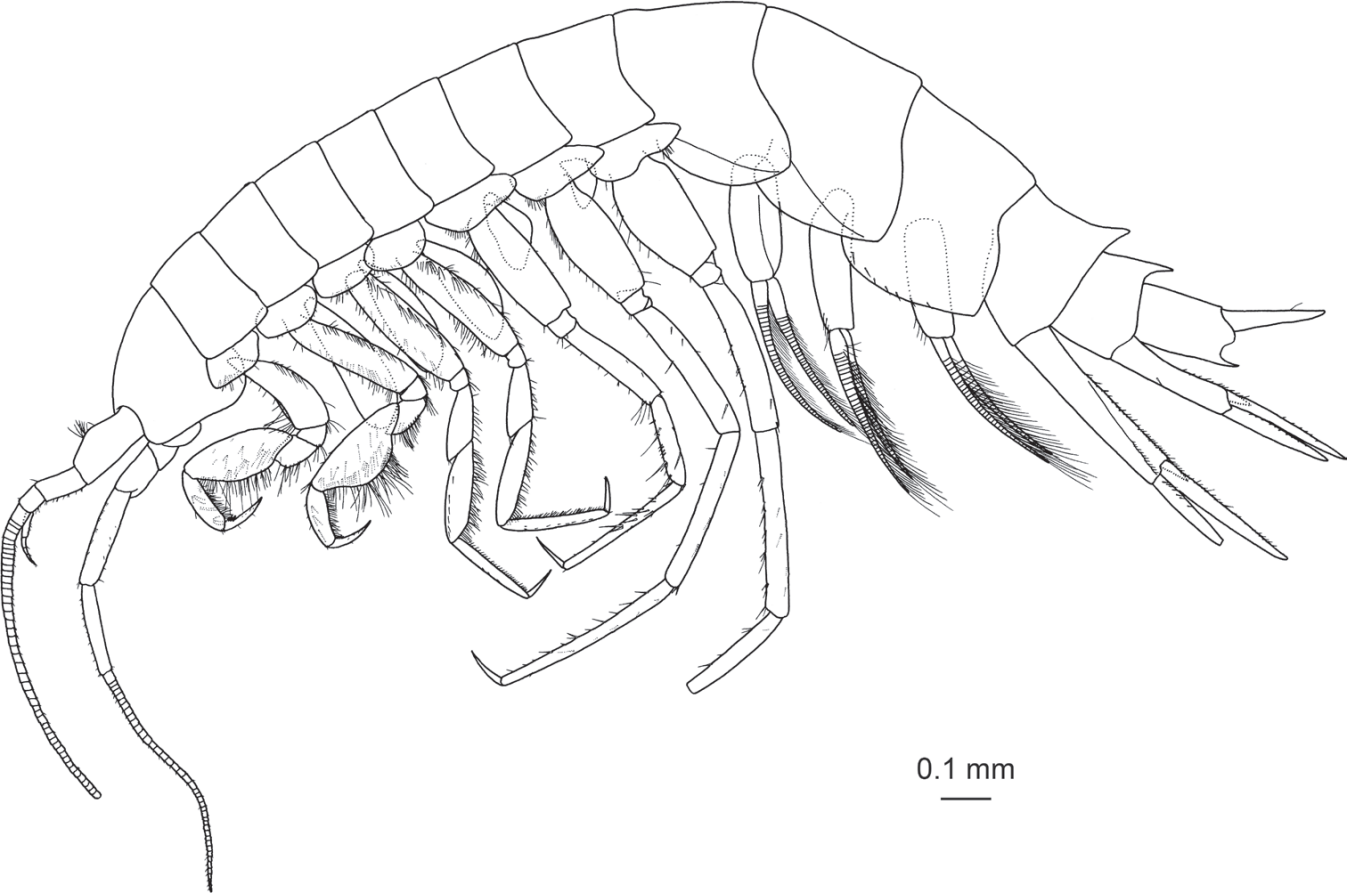
*Princaxeliamarianaensis* Tomikawa & Watanabe, sp. nov., holotype female (BL 23.9 mm). Habitus, lateral view.

**Figure 3.**
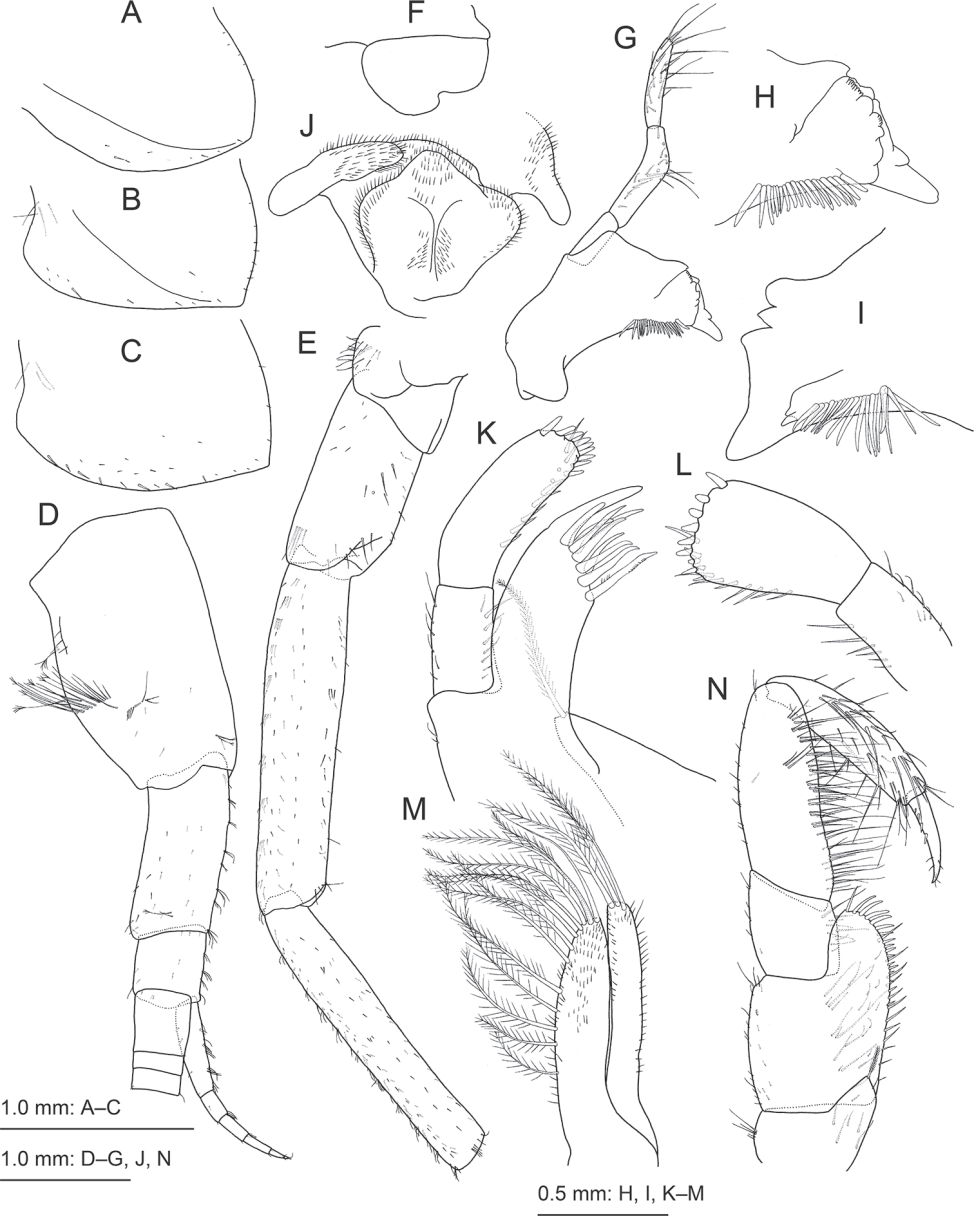
*Princaxeliamarianaensis* Tomikawa & Watanabe, sp. nov., holotype female (BL 23.9 mm) **A** epimeral plate 1, lateral view **B** epimeral plate 2, lateral view **C** epimeral plate 3, lateral view **D** antenna 1, lateral view, some distal articles of primary flagellum omitted **E** antenna 2, lateral view, flagellum omitted **F** upper lip, anterior view **G** left mandible, medial view **H** left mandible, medial view **I** right mandible, medial view **J** lower lip, anterior view **K** maxilla 1, dorsal view **L** palp of maxilla 1, dorsal view **M** maxilla 2, dorsal view **N** maxilliped, dorsal view.

**Figure 4.**
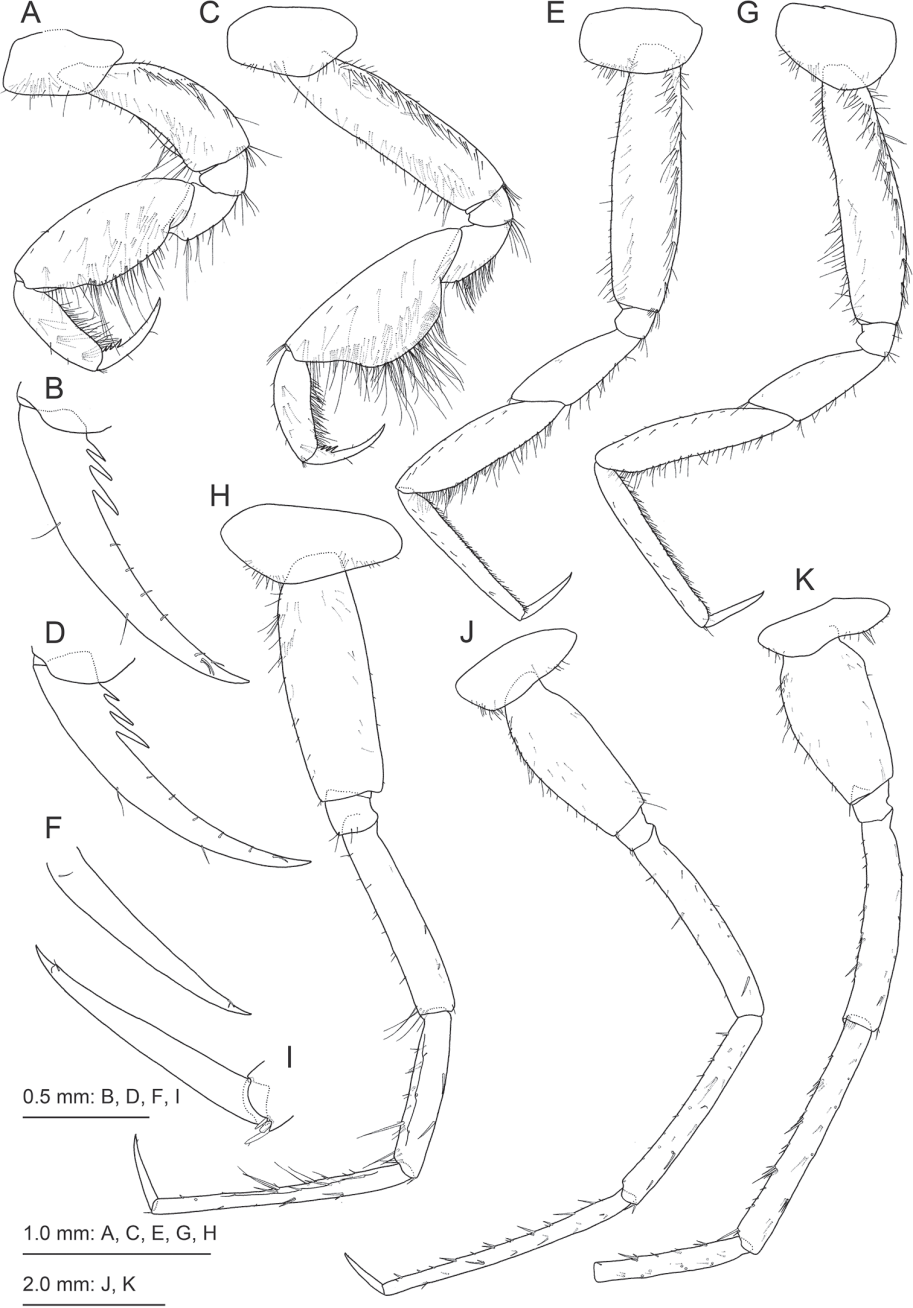
*Princaxeliamarianaensis* Tomikawa & Watanabe, sp. nov., holotype female (BL 23.9 mm) **A** gnathopod 1, lateral view **B** dactylus of gnathopod 1, lateral view **C** gnathopod 2, lateral view **D** dactylus of gnathopod 2, lateral view **E** pereopod 3, lateral view **F** dactylus of pereopod 3, lateral view **G** pereopod 4, lateral view **H** pereopod 5, lateral view **I** dactylus of pereopod 5 **J** pereopod 6, lateral view **K** pereopod 7, lateral view.

**Figure 5.**
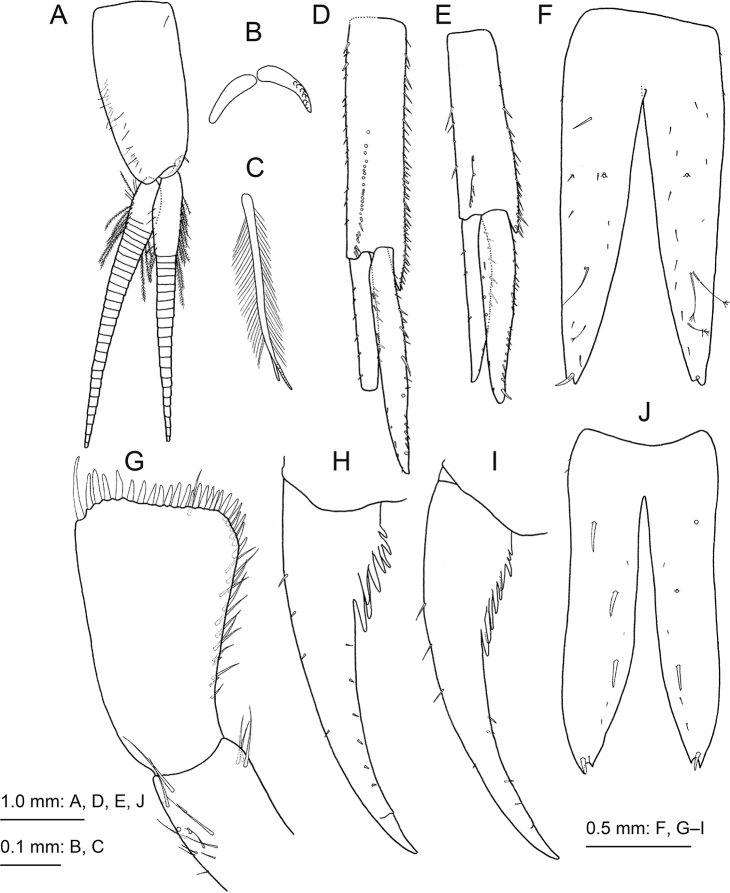
*Princaxeliamarianaensis* Tomikawa & Watanabe, sp. nov., holotype female (BL 23.9 mm) **A** pleopod 1, anterior view, some setae on rami omitted **B** retinacula on peduncle of pleopod 1, anterior view **C** bifid (clothespin) plumose seta on inner basal margin of inner ramus of pleopod 1, anterior view **D** uropod 1, dorsal view, distal part of outer ramus broken **E** uropod 2, dorsal view **F** telson, dorsal view. *Princaxeliajamiesoni* Lörz, 2010, holotype female (BL 56.2 mm) **G** palp of maxilla 1, dorsal view **H** dactylus of gnathopod 1, lateral view **I** dactylus of gnathopod 2, medial view **J** telson, dorsal view.

Figures 2 , 3 , 4 , 5

###### Material examined.

***Holotype***: female (BL 23.9 mm), **AMNH**_IZC 00361360, the surface of the chimney which was named as “Chim 4” in CH 3 site in the Shinkai Seep Field (Okumura et al. 2016), Mariana Trench (11°39.36'N, 143°2.88'W), 5,689–5,683 m, collected by H. K. Watanabe, 17 July 2014.

###### Diagnosis.

Posterodistal corner of epimeral plate 3 quadrate. Primary flagellum article 1 of female antenna 1 not elongate; accessory flagellum article 1 longer than each of the articles 2–6. Maxilla 1 inner plate with 1 terminal plumose seta; palp article 2 expanded, with 8 or 9 apical robust setae. Dactylus of gnathopods 1 and 2 with three strong projections on posterior margin proximal to base. Dorsal margin of coxa 5 highest at proximal end. Venral margin of coxa 7 weakly concave. Telson lobe uniformly tapering distally.

###### Description

**(female). *Head*** (Fig. 2) as long as pereonites 1 and 2 combined; rostrum short, pointed; lateral cephalic corner rounded; eyes absent. Pleon (Fig. 2) with dorsal surfaces of pleonites 1–3 smooth; epimeral plates 1–3 (Fig. 3A–C) with setae on ventral submargin and posterior margin; posterodistal corner of epimeral plates 2 and 3 quadrate. Dorsal margin of urosomites 1 and 2 (Fig. 2) with distally oriented projection.

***Antenna 1*** (Fig. 3D) length 0.3 times BL (distal part broken off); peduncular articles 1–3 with length ratio 1.0 : 0.7 : 0.3; peduncular article 1 broadened, with anterolateral cluster of setae, some weakly plumose; posterior margin of peduncular articles 2 and 3 with clusters of short setae; primary flagellum article 1 length 1.2 times width, 3.0 times as long as article 2; accessory flagellum 6-articulated, article 1 0.9 times as long as articles 2–6 combined; primary flagellum with at least 47 articles.

***Antenna 2*** (Fig. 3E) length 0.4 times BL; anterior margin of peduncular article 2 with setae; peduncular articles 4 and 5 with clusters of short setae on anterior margin, article 4 1.1 times longer than article 5; flagellum with 42 articles.

***Upper lip*** (Fig. 3F) asetose, with asymmetrically incised ventral margin. Mandibles (Fig. 3G–I) slightly asymmetric, incisor margins broad, anteroventral corner with strong tooth; left lacinia mobilis (Fig. 3H) broad, about 0.7 times as long as incisor, multi-dentate; right incisor (Fig. 3I) with three teeth on proximal to anterodorsal corner; right lacinia weak, with two teeth; accessory setal row of left and right mandibles each with about 20 robust setae; molar absent; mandibular palp 3-articulated with length ratio 1.0 : 1.7 : 1.5; article 1 asetose; article 2 posteriorly reflected, articles 2 and 3 with 18 and 22 setae, respectively. Lower lip (Fig. 3J) with broad outer and distinct inner lobes. Maxilla 1 (Fig. 3K, L) with inner and outer plates and palp; inner plate small with apical plumose seta; outer plate subrectangular, with 9 robust apical setae and strong projection; palp 2-articulate; article 1 with marginal setae; article 2 expanded distally with nine and eight robust setae on apical margin of left and right maxilla 1, respectively, and with apical submargin and medial margin lined with setae. Maxilla 2 (Fig. 3M) with inner plate bearing row of 13 plumose setae along apical to medial margin; outer plate slightly longer than inner plate, with three apical plumose setae. Maxilliped (Fig. 3N) with inner and outer plates and palp; inner plate small, subtriangular, not reaching base of palp, with plumose apical seta and short subapical seta; outer plate oval, reaching base of article 2 of palp, with setae along apical to medial margin; palp 4-articulate, long: article 2 longest with inner marginal rows of setae, article 3 with clusters of setae on dorsal and ventral faces and medial marginal setae, and article 4 slender, with robust setae on medial margin.

***Gnathopod 1*** (Fig. 4A, B) coxa subrectangular, length 1.8 times width, ventral margin straight, posterior submargin and medial face with setae; basis arched, with anterior and posterior margins with numerous setae in a row; posterior margin of merus with sparse setae; carpus oval, length 2.5 times width, posterior margin and medial face setose; propodus slender, length 0.6 times that of carpus, posterior margin weakly convex with setae; dactylus slender, slightly curved, posterior margin with three strong projections proximal to base. Gnathopod 2 (Fig. 4C, D) coxa tapering anteriorly, length 1.8 times width, posterior submargin with setae; basis slender and straight, anterior and posterior margins densely setose; carpus widely produced posteriorly with numerous long setae, length 2.3 times width; propodus and dactylus similar to gnathopod 1.

***Pereopod 3*** (Fig. 4E, F) coxa weakly rounded ventrally, with submarginal setae; basis long, posterior margin strongly setose; merus, carpus, propodus, and dactylus in length ratio 1.0 : 1.4 : 1.4 : 0.5; posterior margin of propodus lined with short setae. Pereopod 4 (Fig. 4G) similar to pereopod 3, with coxa tapering anteriorly. Pereopod 5 (Fig. 4H, I) coxa subtriangular, dorsal margin highest at proximal end, anterior and ventral submargins with setae; basis length 2.9 times width, with clusters of setae on anterior margin proximal to base, posterodistal corner weakly produced; merus, carpus, propodus, and dactylus in length ratio 1.0 : 0.8 : 1.2: 0.3; carpus and propodus with robust setae on anterior and posterior margins. Pereopod 6 (Fig. 4J) coxa weakly concave; basis length 2.5 times width, posterodistal corner quadrate; merus, carpus, propodus, and dactylus in length ratio 1.0 : 1.0 : 1.2: 0.3. Pereopod 7 (Fig. 4K) coxa weakly concave; basis length 1.9 times width, weakly expanded anteriorly, posterodistal corner quadrate.

***Coxal gills*** (Fig. 2) on gnathopod 2, pereopods 3–6; coxal gills 2–4 elongate, coxal gill 2 longest, its length exceeding the distal part of basis of gnathopod 2, coxal gill 6 shortest.

***Pleopods 1–3*** (Fig. 5A–C) each with paired retinacula (Fig. 5B) on inner distal margin of peduncle, and bifid (clothespin) setae (Fig. 5C) on inner basal margin of inner ramus; rami articles wide and flattened.

***Uropod 1*** (Fig. 5D) peduncle longer than rami, with 14 basofacial setae, distomedial peduncular projection very strong; inner ramus length 0.8 times that of peduncle, outer ramus distally damaged, rami with setal row along medial and lateral margins. Uropod 2 (Fig. 5E) peduncle slightly longer than rami, with four basofacial setae, distomedial peduncular spine shorter than that of uropod 1; inner ramus length 1.2 times that of outer ramus, rami with setal row along medial and lateral margins. Uropod 3 missing (damaged).

***Telson*** (Fig. 5F) length 2.3 times width, with cleft extending 80% its length; lobes tapering distally with facial setae; apex of each lobe shallowly incised with small robust seta.

###### Etymology.

The specific name is an adjective derived from the type locality, the Mariana Trench.

###### DNA sequence.

A single nucleotide sequence of COI was obtained from the holotype (AMNH_IZC 00361360; 658 bp).

###### Remarks.

The morphologies of *P.marianaensis* sp. nov. and congeners are summarized in Table 1. *Princaxeliamarianaensis* sp. nov. is most similar to *P.abyssalis* Dahl, 1959 in having a short first flagellar article of the female antenna 1, a weakly setose maxilla 1, coxa 5 with its dorsal margin highest at the proximal end and its distal margin rounded, and a uniformly tapering telson. However, *P.marianaensis* sp. nov. differs from the description of *P.abyssalis* in having the posterodistal corner of epimeral plate 3 quadrate in *P.marianaensis* sp. nov. but rounded in *P.abyssalis*; the accessory flagellum article 1 of the female antenna 1 longer than each of the articles 2–6 in *P.marianaensis* sp. nov. but equal to the length of the remaining segments in *P.abyssalis*; and the ventral margin of the coxa 7 weakly concave in *P.marianaensis* sp. nov. but straight in *P.abyssalis*.

**Table 1.** Morphological comparison of *Princaxelia* species.

	*P.marianaensis* Tomikawa & Watanabe, sp. nov.	*P.abyssalis* Dahl, 1959	*P.jamiesoni* Lörz, 2010	*P.magna* Kamenskaya, 1977	*P.stephenseni* Dahl, 1959
Maximum body size	female 23.9 mm	male 21 mm, female 32 mm	male 57 mm, female 61 mm	male 52 mm	male 10 mm, female 11 mm
Epimeral plate 3 posterodistal corner	quadrate	rounded	quadrate	quadrate	weakly rounded
Dorsal projections on urosomites 1 and 2	pointing toward distal end	unknown	pointing toward distal end	pointing upright	pointing toward distal end
Upper lip	strongly asymmetrical	unknown	slightly asymmetrical	strongly asymmetrical	nearly asymmetrical
Maxilla 1 palp article 2	expanded	expanded	expanded	expanded	not expanded
Maxilla 1 palp article 2	9 apical robust setae	less than 14 apical robust setae	25 apical robust setae	approx. 10 apical robust setae	7 apical robust setae
Maxilla 1 inner plate	1 plumose seta	1 plumose seta	1 plumose seta	6 plumose setae	1 plumose seta
Female antenna 1 primary flagellum article 1	not elongated	not elongated	elongated	unknown	elongated
Female antenna 1 accessory flagellum article 1	longer than each of the rest	equal to length of remaining articles	longer than each of the rest	unknown	unknown
Gnathopods 1 and 2 dactyli	3 strong projections near the base	unknown	8–9 strong projections near the base	4 strong projections near the base	unknown (absent?)
Coxa 5 dorsal margin	highest at proximal end	highest at proximal end	straight	convex	straight / convex
Coxa 5 distal margin	rounded	rounded	rounded	slightly pointed	straight
Coxa 7 ventral margin	shallowly concave	straight	slightly concave	slightly concave	straight
Telson lobe	uniformly tapering distally	uniformly tapering distally	tapering from distal 1/3	weakly tapering distally	unknown
References	This study	Dahl (1959)	Lörz (2010); this study	Kamenskaya (1977)	Dahl (1959); Lörz (2010)

*Princaxeliajamiesoni* Lörz, 2010 was described from 7,703 m and 9,316 m in the Japan and Izu-Ogasawara trenches, respectively (Lörz 2010), and subsequently from 7,055–9,583 m in the Kurile-Kamchatka Trench (Jażdżewska and Mamos 2019). Examination of the holotype of *P.jamiesoni* reveals new features not originally described which facilitate differentiation of this species from *P.marianaensis* sp. nov.: the palp article 2 of the maxilla 1 bears eight or nine robust apical setae in *P.marianaensis* sp. nov. but 25 robust apical setae in *P.jamiesoni* (Fig. 5G); the dactylus of gnathopods 1 and 2 has three strong projections proximal to its base in *P.marianaensis* sp. nov., but eight or nine strong projections proximal to the base of the dactylus in *P.jamiesoni* (Fig. 5H, I); and the telson lobe uniformly tapers distally in *P.marianaensis* sp. nov. but tapers from the distal 1/3 in *P.jamiesoni* (Fig. 5J). While two projections on the dactylus of the left gnathopod 2 were originally described for *P.jamiesoni*, we report nine projections on the right gnathopod 2 of the holotype; we believe that Lörz (2010) described the damaged left gnathopod 2.

**Figure 6.**
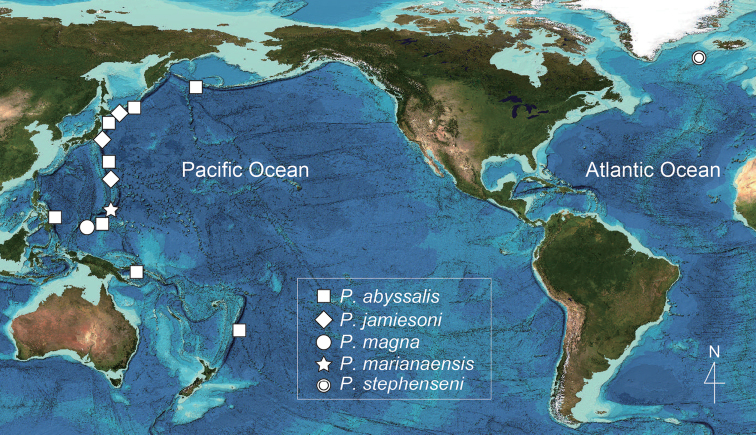
Geographical distributions of the species of *Princaxelia* (map data from GEBCO Compilation Group [2020]). The exact location of the distribution of *P.abyssalis* in the Aleutian Trench is uncertain.

The morphology of *Princaxelia* is consistent with an animal that swims in that its body is streamlined, flat, and has well-developed pleopods (Lörz 2010). Analyses of the locomotion of *Princaxelia* species demonstrate they have a high swimming ability – a trait useful for preying on other amphipods in hadal trenches (Jamieson et al. 2012). Amphipods lack a planktonic larval stage and generally have low dispersal ability (Chapman 2007). Judging from known habitat depths of *Princaxelia*, with the exception of the bathypelagic *P.stephenseni*, the distributions of species might be expected to be restricted to individual trenches. However, *P.abyssalis*, and especially *P.jamiesoni*, are reported from multiple trenches (Fig. 6) (Kamenskaya 1981, 1997; Lörz 2010; Jażdżewska and Mamos 2019). Deep-sea amphipod species previously regarded as widely distributed have since been found to contain cryptic species (e.g., Narahara-Nakano et al. 2018). Lörz (2010) also considered that *P.abyssalis*, as reported from multiple trenches by Kamenskaya (1981), may contain other or undescribed species. It is possible that *P.abyssalis* and *P.jamiesoni* represent species complexes, but a greater understanding of species diversity of this hadal-dwelling genus will require additional genetic and morphological analyses.

### Key to species of *Princaxelia* modified from Lörz (2010)

We added *P.marianaensis* sp. nov. to the key by Lörz (2010) and modified the key to include the characteristics of the telson, which was not considered by Lörz (2010).

**Table:** 

1	Palp article 2 of maxilla 1 expanded	**2**
–	Palp article 2 of maxilla 1 not expanded	***P.stephenseni* Dahl, 1959**
2	Inner plate of maxilla 1 with 1 terminal plumose seta	**3**
–	Inner plate of maxilla 1 with several plumose setae	***P.magna* Kamenskaya, 1977**
3	Primary flagellum article 1 of female antenna 1 not elongate; dorsal margin of coxa 5 highest at proximal end; telson lobe uniformly tapering distally	**4**
–	Primary flagellum article 1 of female antenna 1 elongate; dorsal margin of coxa 5 straight; telson lobe tapering from distal 1/3	***P.jamiesoni* Lörz, 2010**
4	Posterodistal corner of epimeral plate 3 rounded; accessory flagellum article 1 of female antenna 1 equal to length of remaining articles; ventral margin of coxa 7 straight	***P.abyssalis* Dahl, 1959**
–	Posterodistal corner of epimeral plate 3 quadrate; accessory flagellum article 1 of female antenna 1 longer than each of remaining articles; ventral margin of coxa 7 weakly concave	***P.marianaensis* Tomikawa & Watanabe, sp. nov.**

## Supplementary Material

XML Treatment for Princaxelia marianaensis
